# Unsupervised and supervised learning with neural network for human transcriptome analysis and cancer diagnosis

**DOI:** 10.1038/s41598-020-75715-0

**Published:** 2020-11-05

**Authors:** Bo Yuan, Dong Yang, Bonnie E. G. Rothberg, Hao Chang, Tian Xu

**Affiliations:** 1 Westlake Institute for Advanced Study, Westlake University, Hangzhou, China; 2 Department of Genetics, Yale Cancer Center, Howard Hughes Medical Institute, Yale University School of Medicine, 295 Congress Avenue, New Haven, CT 06510 USA; 3 Zhiyuan College, Shanghai Jiao Tong University, Shanghai, China; 4 Medical Oncology, Department of Internal Medicine, Yale Cancer Center, Yale University School of Medicine, New Haven, USA; 5 Present Address: Deptartment of Cell Biology, Harvard Medical School, Boston, MA 02138 USA

**Keywords:** Cancer models, Machine learning

## Abstract

Deep learning analysis of images and text unfolds new horizons in medicine. However, analysis of transcriptomic data, the cause of biological and pathological changes, is hampered by structural complexity distinctive from images and text. Here we conduct unsupervised training on more than 20,000 human normal and tumor transcriptomic data and show that the resulting Deep-Autoencoder, DeepT2Vec, has successfully extracted informative features and embedded transcriptomes into 30-dimensional Transcriptomic Feature Vectors (TFVs). We demonstrate that the TFVs could recapitulate expression patterns and be used to track tissue origins. Trained on these extracted features only, a supervised classifier, DeepC, can effectively distinguish tumors from normal samples with an accuracy of 90% for Pan-Cancer and reach an average 94% for specific cancers. Training on a connected network, the accuracy is further increased to 96% for Pan-Cancer. Together, our study shows that deep learning with autoencoder is suitable for transcriptomic analysis, and DeepT2Vec could be successfully applied to distinguish cancers, normal tissues, and other potential traits with limited samples.

## Introduction

Deep learning with artificial neural networks has recently surpassed traditional approaches, and the performance of trained specialists in handling massive and complex data in multiple areas. For a range of tasks, particularly in image recognition^1^ and text mining^2^, deep learning based artificial intelligence outperforms humans because of its objective data handling and successful high-level feature extraction^1–5^. Indeed, the approach has also been successfully employed for a number of biomedical analyses, including mutation detection^6^ and morphological classifications for fMRI data^7^, tumor histopathological or photographic images^4,5,8^, and eye diseases^9^.

Cancers or other pathological and biological processes are largely depended on gene activity or gene expression. Biomarkers using individual gene expression or mutation information have shown predictive power in diagnosis or therapy for several diseases^10–13^. Systematic and quantitative measures of gene expression by microarray and next-generation sequencing, i.e., transcriptomics, could significantly facilitate research, diagnosis, and therapeutics. However, the collective expression status of the genes, the transcriptome, in the genome of a specific type of cell or tissue is large and complex, making it difficult to analyze and conceptualize for research and application. Since the beginning of the millennium, a number of studies have attempted to employ different machine learning methods to characterize gene expression for several cancer types^12,14–18^. These studies illuminated a new path for analyzing transcriptomic data. However, they were limited by small sample size, biomarker selection, or narrow model generalizability. Furthermore, the approaches of linear transformation in these studies are used to extract complex features, which restricts the model from handling linearly inseparable data.

The idea that deep learning could be applied to transcriptomic analysis is tantalizing. However, because transcriptomes do not share similar intrinsic characteristics or structure with images or text, such as local correlation^1,19^, or sequential structure^20^, convolutional neural networks for analyzing imaging and language are not particularly suitable for analyzing transcriptomic data. Extracting meaningful or key features from complex transcriptomic data remains a daunting challenge. It remains uncertain whether deep learning could be used to reduce the dimensionality and complexity of transcriptomic data and extract characteristic features for predicting biological and pathological behavior such as cancer diagnosis.

Here we report the construction and training of an unsupervised deep autoencoder to extract features of high dimensional transcriptomic data from a large collection of normal human tissues and of tumor samples of different types. We show that the extracted transcriptomic features have retained adequate information such that they could be decoded back to original gene expression profiling. Furthermore, when coupled those extracted features with the question to separate different cancer or normal tissue samples. Combined with supervised classification, the extracted features can be effectively used to distinguish normal samples of different tissue origins and successfully diagnose tumors in Pan-cancer or tissue-specific cancer analyses.

## Results

### Unsupervised feature extraction of transcriptome with deep autoencoder

In order to develop a deep neural network to learn features from human transcriptomic data, we collected gene expression data from various types of human tumors and corresponding normal tissues from the “Gene Expression Across Normal and Tumor Tissue” database (GENT, Affymetrix U133plus2 platform), which collects the majority of the published tumor microarray data from NCBI Expression Omnibus^21,22^. The total data set consisted of 20,654 individual microarrays, 17,258 tumors and 3396 normal tissue samples. The original dataset were normalized and preprocessed to remove outliers and duplicates, in the end, there were n = 16,532 tumors and n = 3202 normal tissue samples were then used in subsequent analyses.

To guard against overfitting the data with the available sample size, the training was conducted using a reduced gene set consisting of 978 Landmark Genes (L1000)^23^ from each sample. As gene expression is highly correlated, this set of Landmark Genes has been reported to represent approximately 80% of the information within the transcriptome^24^ and multiple studies have demonstrated their applicability^25–27^.

**Figure 1:**
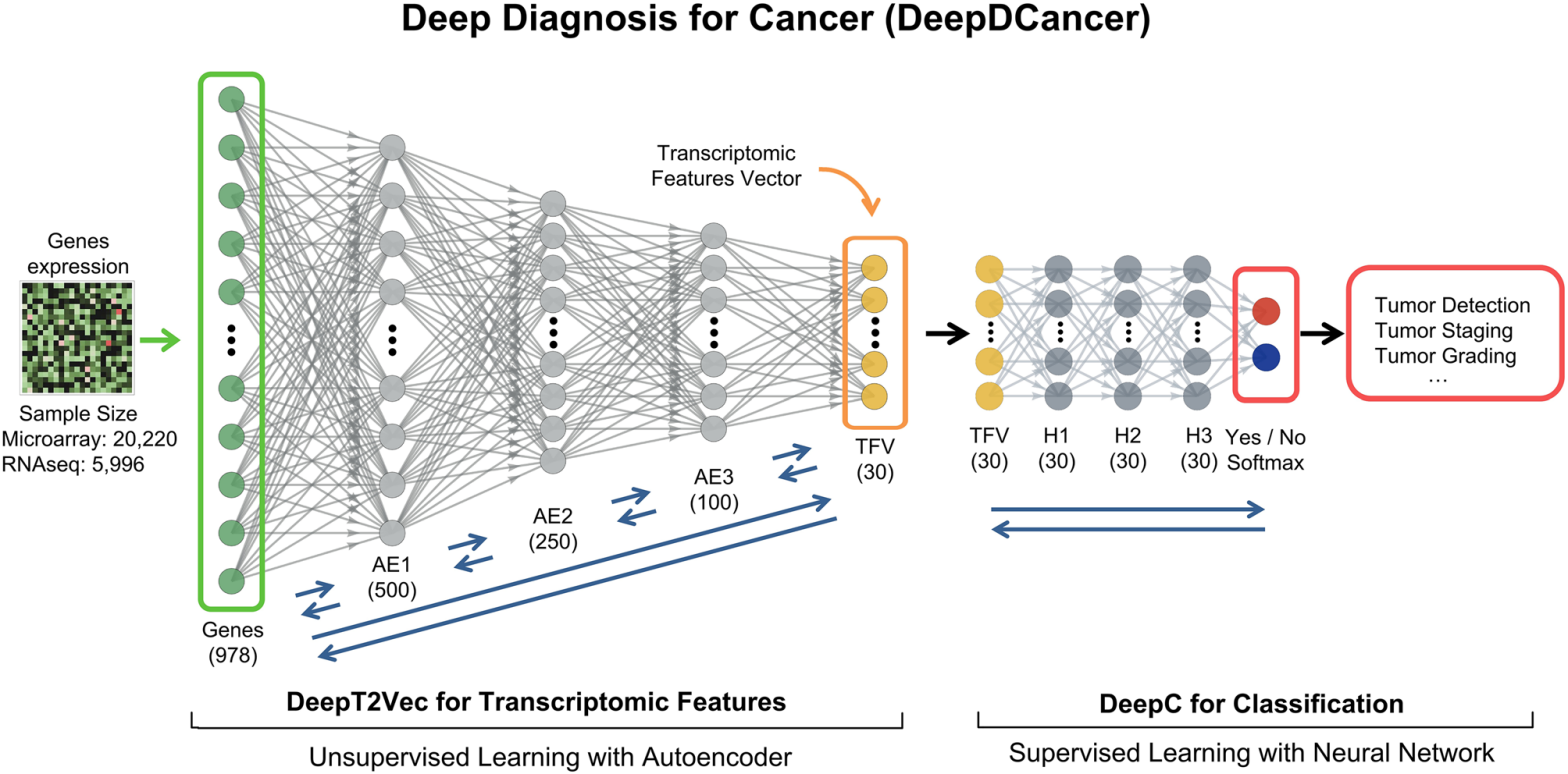
Deep Diagnosis neural network for transcriptomic data analysis and cancer diagnosis. A deep neural network termed Deep Diagnosis for Cancer (DeepDCancer) was constructed to extract transcriptomic features and to classify normal and tumor samples. DeepT2Vec, a 5-layer Deep Autoencoder (AE), was trained in an unsupervised fashion to extract informative features from high dimensional gene expression data (978 L1000 Landmark genes) and to compress them into a 30-dimensional Transcriptomic Feature Vector (TFV). The TFVs from samples of known classes were subsequently used in supervised training on a fully connected Deep Softmax Classifier (DeepC) with three hidden layers (H) to distinguish normal samples from tumors or tumors at different grades and stages. Human gene expression data from 20,220 samples of normal or tumor tissues were used in the analysis.

A deep neural network termed Deep Diagnosis for Cancer (DeepDCancer) was constructed with two parts: (1) DeepT2Vec (Deep Transcriptome to Vector): that extracts transcriptomic features and (2) DeepC to classify normal and tumor samples (Fig. 1). For extracting features from the human transcriptome, we used the data to train a deep autoencoder, a specialized deep neural network, in an unsupervised fashion, i.e., no manual annotation or labels have been used in training^1^. A deep autoencoder processes the data through successive layers of progressively fewer nodes (neurons), by extracting features to compress highly complex information into low dimensional space^1^. We designed our autoencoder, DeepT2Vec, with five layers, each yielding a 30–50% reduction in neurons (Fig. S1). We then performed unsupervised training for this deep autoencoder, which allows the extraction of transcriptomic features in an unbiased fashion (“Materials and methods”).

The Stochastic Gradient Descent (SGD) algorithm was used to minimize the reconstruction error during training. The hyperparameters (learning rate, batch size, and epoch) were adjusted to optimize the training (“Materials and methods”). 10% of the microarray data (1639 tumors and 334 normal samples) were randomly selected and kept aside as the test dataset, while the remaining 90% data (14,893 tumors and 2868 normal samples) were referred as the experimental dataset. In the unsupervised training, 70% of the experimental data were used for training the deep autoencoder, while the rest of the 30% experimental data were used as a validation dataset to monitor the training process and to avoid overfitting. DeepT2Vec allows us to embed the high-dimensional gene expression data from each sample into a continuous 30-dimensional vector termed the Transcriptomic Feature Vector (TFV) (Fig. 1).

**Figure 2:**
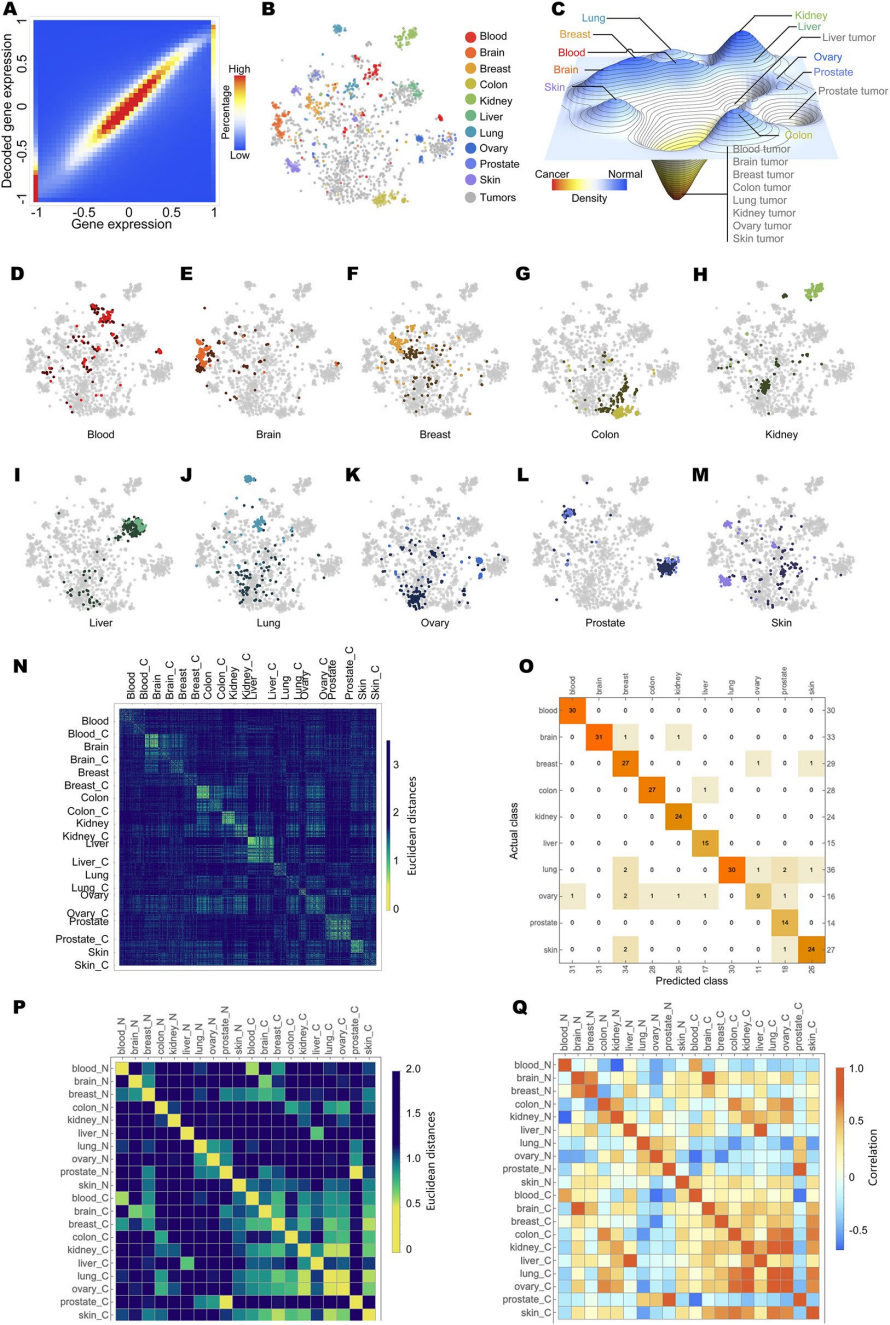
The success of DeepT2Vec transcriptomic feature extraction revealed by expression recapitulation and TFV distribution. (**A**) gene expression values decoded from DeepT2Vec generated TFVs were compared to their original data (R^2^ = 0.7). TFVs from samples of normal tissues (color dots) and tumors (grey dots) generated by DeepT2Vec were visualized by t-Distributed Stochastic Neighbor Embedding (t-SNE) dot plot (**B**) and t-SNE density plot (**C**). (**D**–**M**) TFVs from tumors of individual tissues (darker color) and corresponding normal samples (light color) were visualized with t-SNE. **(N)** Pairwise Euclidean distances between TFVs learned from the same tissues of origin or corresponding tumors, respectively. **(O)** A classifier trained on the TFVs in order to separate different normal tissues demonstrates high predictive power (overall accuracy 0.917). **(P)** Euclidean distances and **(Q)** correlations between the centroids of TFV for any type of normal tissues and tumor samples.

To assess the effectiveness of DeepT2Vec in extracting informative features from human transcriptomic data, we applied DeepT2Vec to the test dataset to examine its ability to encode the gene expression data into TFVs and then to decode them back. We found that DeepT2Vec can successfully extract the transcriptomic features and decode to recapitulate the original data (Fig. 2A). Our results demonstrate that the features extracted by DeepT2Vec are informative, and it can be used to convert complex transcriptomic data into low dimensional vectors for comparison and analysis.

Differences in tissue identities are derived from distinctive transcriptional activities or gene expression profiles. A successful DeepT2Vec should extract representative transcriptomic features demonstrating the similarity of TFVs within a given tissue and diversity of TFVs between different tissues. We used the TFVs generated by DeepT2Vec from the test dataset and visualized the resulting TFVs of normal samples from different tissues with unsupervised t-Distributed Stochastic Neighbor Embedding (t-SNE)^28^. T-SNE embeds the 30-dimensional TFVs data into a two-dimensional space, where similarities between TFVs are displayed according to the proximity of locations. Among normal tissues, the resulting TFVs displayed characteristic clusters for each tissue type and separation of clusters for samples from different tissues, indicating that DeepT2Vec has successfully extracted transcriptomic features representative of these tissues (Fig. 2B, color dots).

We next examined the TFVs from tumor samples with unsupervised t-SNE. Interestingly, the majority of tumor samples irrespective of their tissues of origins not only segregated from the normal clusters but also coalesced into a common area (Fig. 2B, gray dots), which can be best visualized in a density t-SNE landscape (Fig. 2C). Within each organ system, the separation of tumors from the corresponding normal samples was also evident (Fig. 2D–M). Together, these data show that DeepT2Vec, in an unsupervised fashion, has successfully learned features of the human transcriptome that can distinguish tumors from normal tissues. Additionally, we calculated the pairwise Euclidean distances between TFVs learned from the same tissues of origin or corresponding tumors, respectively (Fig. 2N). Consistent with the t-SNE plots, we observe a substantial similarity between each sample from the same tissue or cancer types, respectively and demonstrate the distinction between different data groups, normal tissues in particular (average in-group distance 1.46 ± 0.23, inter-group distance 1.93 ± 0.35, Mann–Whitney test p-value < 1e−10, ANOVA p-value < 1e−10). As we observed from the t-SNE plots, most cancer samples (except for prostate cancer) are topologically close to each other (the average in-group distance for cancer 1.86 ± 0.38, the inter-group distance for normal tissues 1.91 ± 0.40, Mann–Whitney test p-value < 1e−10, ANOVA p-value < 1e−10). For 10 out of 10 tissue-tumor pairs, the ANOVA tests demonstrate the significant differences between each pair with p-values < 1e−5, with stringent Bonferroni’s correction. Furthermore, a classifier trained on the TFVs in order to separate different normal tissues demonstrates high predictive power (Fig. 2O, overall accuracy 0.917). Last but not least, we calculate the centroid TFV for each of the data groups and compare the distance and correlation between them (Fig. 2P,Q), which shows consistent results with the sample-level analysis.

These results that tumors of different tissue origins have a shared profile of transcriptomic features are particularly interesting. Although it is known that cancers share common biological properties such as uncontrolled growth, there has been no molecular or simple quantitative measure for “cancerousness”. Our results not only suggest a common underlying molecular mechanism of tumorigenesis but also provide a transcriptome-based quantitative measurement that could be developed for research and therapeutic applications.

### Cancer diagnostic prediction with DeepT2Vec and supervised classifier

**Figure 3:**
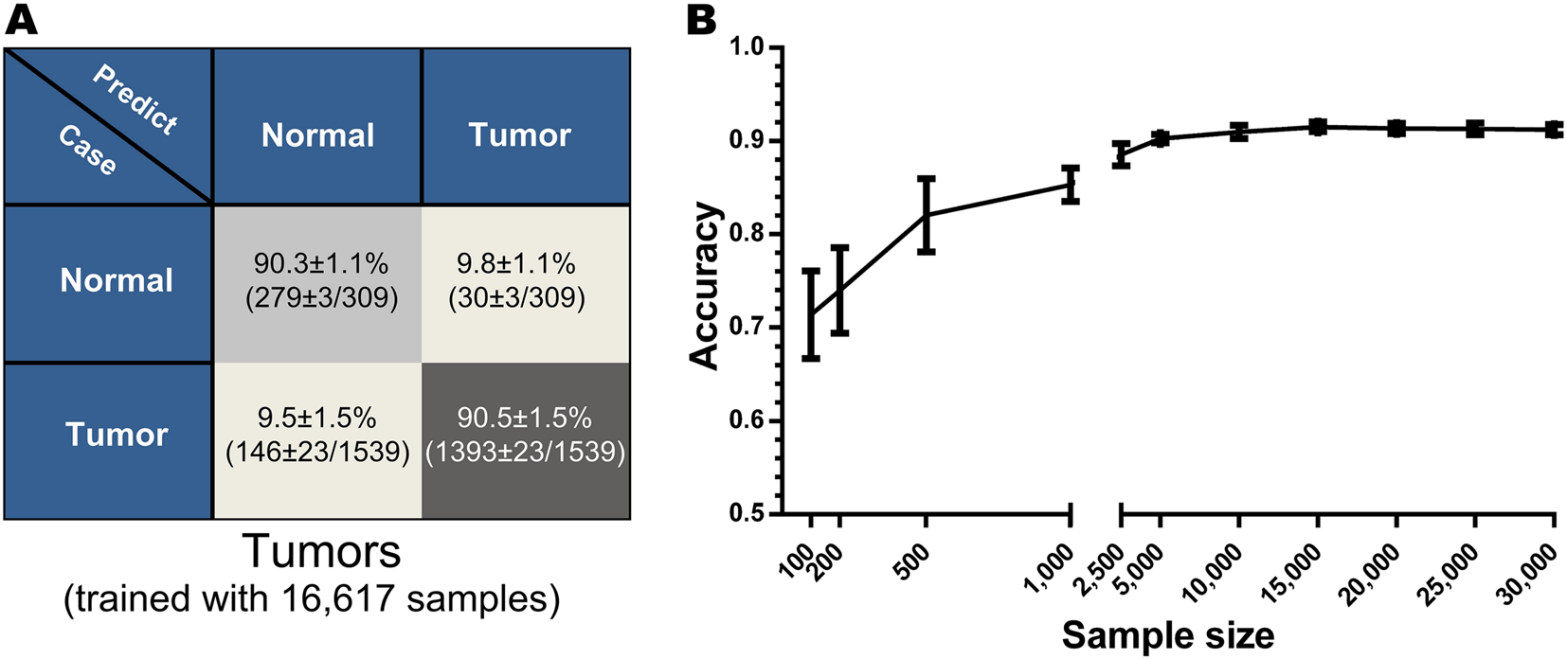
Successful Pan-Cancer diagnosis by DeepDCancer neural network. (**A**) Diagnosis of tumors versus normal samples with mixed tissue origin by DeepDCancer is shown in the confusion matrix. 10 fold validations were performed, and the accuracy is illustrated with mean $\pm$ standard deviation. (**B**) The performance of DeepDCancer trained under different sample sizes is illustrated. With the increasing size of training datasets, the accuracy for diagnosis increased logarithmically first and reached above 90% with as few as 5000 samples.

The unsupervised DeepT2Vec has successfully generated a characteristic TFV for every sample. One of the potential applications could be using those features for supervised classification, as we tested in Fig. 2O to separate tissue types. Given that TFVs for either normal or tumor samples have projected into distinctive space in t-SNE, these TFVs (d = 30) should allow us to distinguish tumors from normal tissues, too. To test this, we constructed a classifier to produce diagnostic predictions. We, therefore, trained a softmax classifier (DeepC) using supervised or labeled data in the latent space, i.e., the feature space (d = 30). DeepC consisted of the TFV input layer, three fully connected inter-layers, and a Softmax output layer, which is logistic regression for classification (Fig. 1A, right panel). For the supervised training, 90% of the experimental dataset with original clinical annotations were used to generate the normal versus tumor classifier, while the remaining 10% of the experimental data were used for validation. Up-sampling was used to avoid data imbalance. The parameters of DeepC were optimized with the SGD algorithm, and 10-cross-validation was applied to avoid overfitting^19,29^. After the generation of DeepC for the Pan-Cancer classification, the test dataset was used for evaluation. Our data showed that, using the 30-d feature vectors only without prior knowledge and manual gene set curation, the classifier correctly identified more than 90% of the tumor and normal samples (Fig. 3A).

In order to assess the effect of sample size on the performance of DeepC, we conducted sensitivity analyses, where we carried out similar supervised training with different sizes of experimental samples (“Materials and methods”). Classification accuracy was improved significantly as sample size increased initially, reaching above 90% at 5000 samples, and then plateaued with a further increase of sample size (Fig. 3B). Interestingly, there was substantial accuracy even when the sample size was relatively small (Fig. 3B, 85% at 1000 samples).

**Figure 4:**
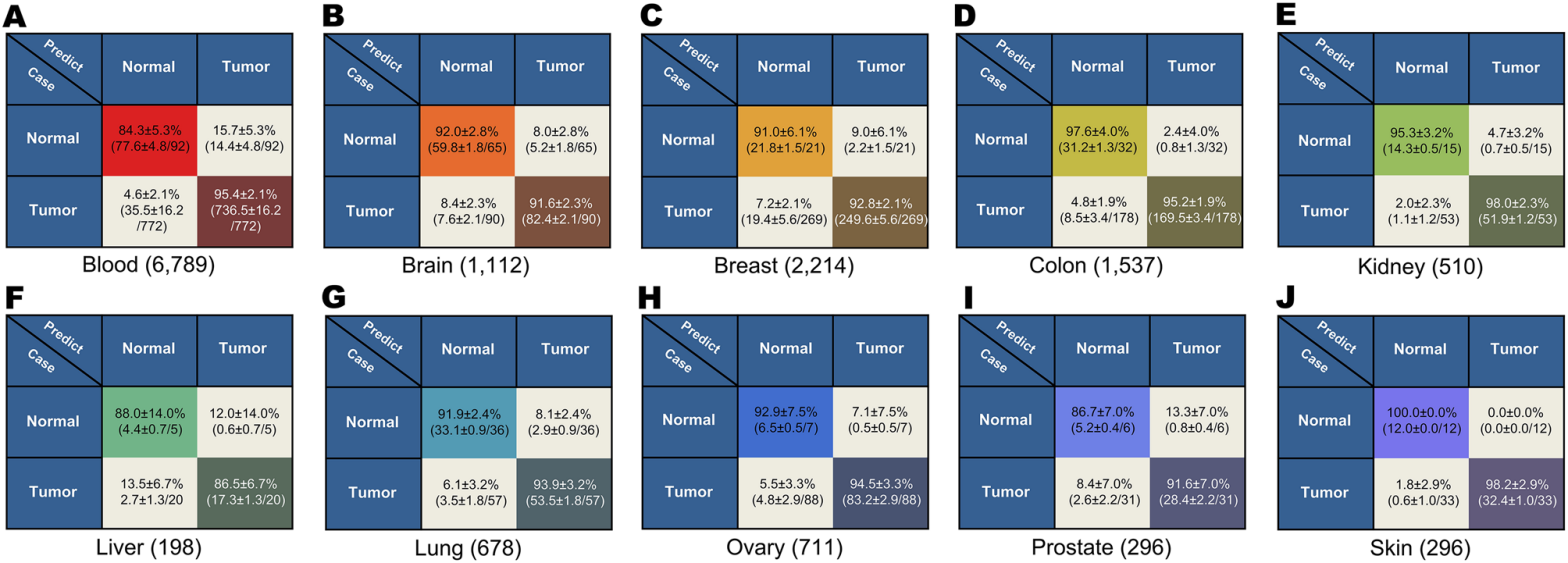
Successful diagnosis for tumors of different tissue origins. Diagnostic predictions of tumor versus normal samples by DeepDCancer for individual tissues are shown in matrices. 10 fold validations were performed, and the accuracy is illustrated with mean $\pm$ standard deviation. The sample sizes used in training are indicated in parentheses.

The training and prediction of the classifier described above used a mix of tumors from different tissues. However, tumor diagnosis and classification are traditionally conducted based on their tissue of origin. Given that the TFVs for different tissues are well clustered and the fact that our approach could yield significant accuracy with a relatively small sample size, we conducted separate classifier training for tumors from individual tissue origins and the corresponding normal tissues (e.g., DeepC-brain for tumors and normal samples from brain). Tumor samples used in training were from brain, colon, breast, kidney, lung, skin, liver, prostate, ovary, and blood (“Materials and methods”). Again, TFVs generated from DeepT2Vec were used as inputs, and 90% of the experimental data for each tumor type were used for training, while 10% of the experimental data were used in 10-cross-validation excises with SGD for optimization. The resulting individual classifiers were then used on the test dataset for individual types of tumors. The accuracies for individual tumor classifications range from 86% for liver to 98% for skin (Fig. 4). While the accuracy for liver tumors is lower than Pan-Cancer prediction, the other classifications for individual tumor types were improved, which is consistent with the notion that samples from the same tissue share more transcriptomic features than those from different tissues. Our results support the traditional practice of classification of tumors according to their tissue of origin.

We also compared other state-of-the-art methods for dimensionality reduction and its predictive power with DeepDCancer (Fig. S2). Principal components analysis (PCA) and non-negative matrix factorization (NMF) are two of the most powerful tools that have been broadly used in gene expression analysis. However, they can only capture linear features, so we also compare the model with nonlinear PCA (with a linear kernel and an RBF kernel, respectively). Our results show that, with dimensionality reduction, gene expression analysis can be constructed in a comparatively low dimensional space with the biological characteristics retained. Then in the low dimensional space, classification models can be trained with higher data efficiency, i.e., the minimal sample size required. Although nonlinear methods, in general, take longer time to compute (PCA/NMF: < 1 min; kernel PCA and DeepDCancer: > 30 min), the potential to capture the nonlinear information from the cell system is still plausible.

Together these results indicate that DeepDCancer which combines unsupervised training DeepT2Vec with supervised training DeepC can successfully analyze complex transcriptomic data for cancer diagnosis. Furthermore, DeepT2Vec trained with a large dataset has extracted powerful features and successfully achieved dimensionality reduction. It generates highly informative and low-dimensional TFVs that enable the generation of classifiers, DeepCs, with relatively small datasets for distinguishing biological and pathological samples.

### Cancer grading and staging classification

Cancer grading and staging classifications are crucial diagnostic parameters that direct therapeutic practice^30^. In recent years, specific mutations and biomarkers have been associated with specific grades and stages of cancers^31^. Therefore, it is possible that cancers of different grades and stages would display distinctive transcriptomic features, and such features could be extracted using deep learning and used for diagnostic prediction. We, therefore, applied DeepT2Vec to grading and staging data and used the resulting TFVs to train classifiers for cancer grading and staging predictions.

**Figure 5:**
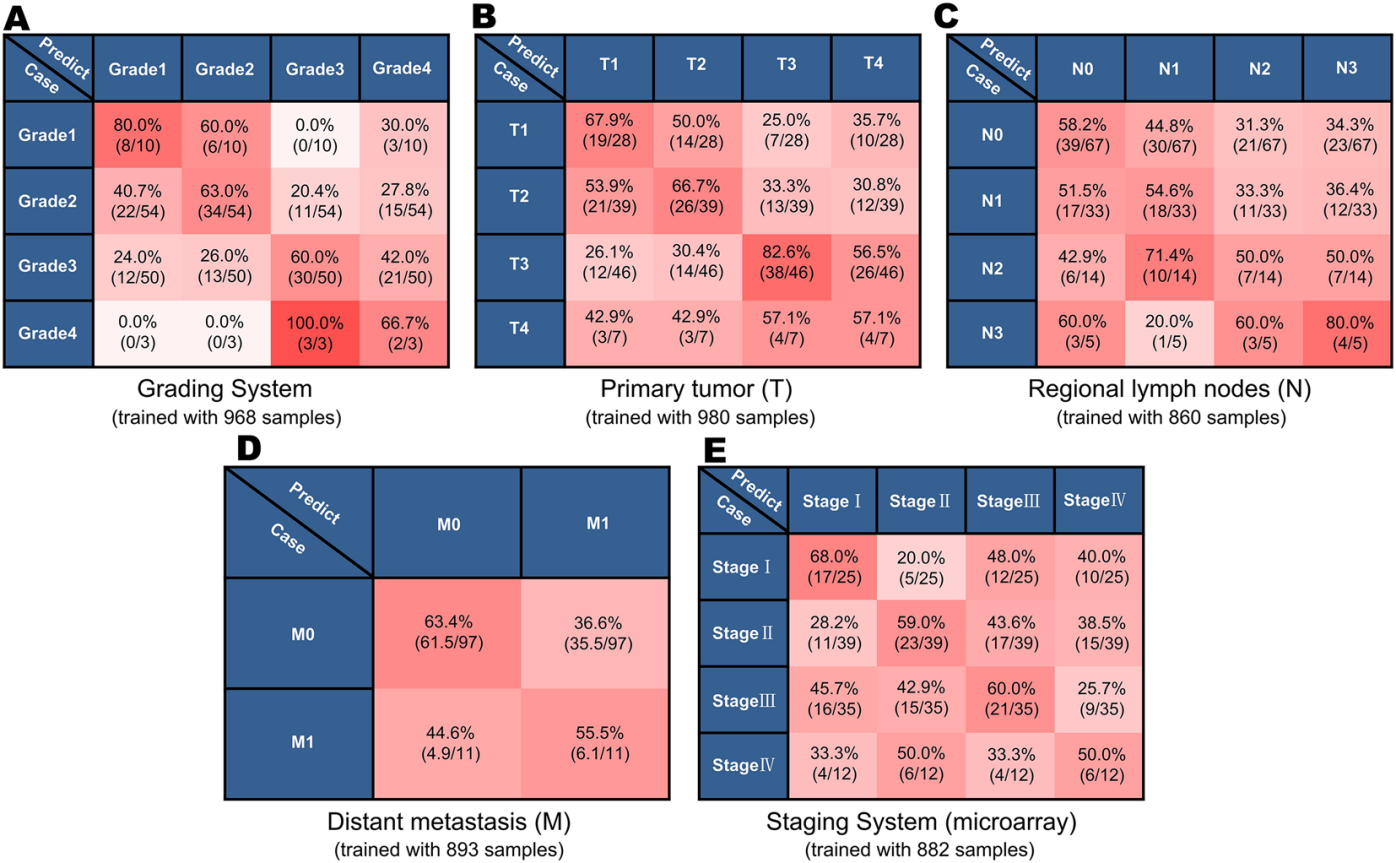
Cancer grading and staging classification by DeepDCancer. Classification predictions for different grades or stages of tumor samples are shown in the corresponding confusion matrices. Training with data from the grading system (**A**) has yielded successful predictions. For the TNM tumor staging system, training with tumors samples with different stages of regional lymph nodes (N) or distant metastasis (M) have also yielded successful predictions (**B**,**C**) while data from different stages of primary tumor content (T) did not (**D**). Predictions for the integrated tumor staging system from microarray (**E**) data exhibit intermedium results.

To achieve this, we used the part of the microarray data set that contains tumor grading or staging information (1274 and 1182 Pan-Cancer samples with grading and staging information, respectively). For classification of cancers at different grades and stages, we employed the one-versus-rest strategy^19^, which involves training a single classifier for each class or each grade or stage of cancers (Fig. 1). Similarly, the samples in the experimental dataset were used for training, while the samples in the test dataset were used for testing (“Materials and methods”). 10 fold cross validation was used during training to avoid overfitting. In testing, each sample or its TFV was then analyzed by these classifiers to find out whether it belongs to a given class (Fig. 5).

Tumor grading is a system that uses the abnormal morphology of tumor cells and tissues under a microscope as an indicator of how quickly a tumor is likely to grow and spread (Grade 1, 2, 3, and 4)^32^. Interestingly, the classifiers, which trained with the tumor samples with grading annotation, generated accurate classification for tumor grade (Fig. 5A). While these results indicate that tumors of different grades undergo gene expression profile shifts and could be used for predicting cancer progression, these predictions were not as robust as the predictions for individual tumor types described above.

We then performed a similar analysis for cancer staging, which focuses on various criteria of physiological and morphological appearance of tumors^33^. The TNM system is the most widely used cancer staging system^30^, in which the T refers to the size of the primary tumor and its extend growing into nearby tissues (T1, T2, T3, and T4). The N refers to whether there is no or different degrees of lymph node metastasis (N0, N1, N2, and N3), while the M refers to whether there are distant metastases or not (M1 and M0). While the prediction performance for T is similar to tumor grading, the identification for N and M were less robust (Fig. 5B–D). As expected, the classification for integrated cancer staging data is a combined outcome of T, N, and M (Fig. 5E).

It is exciting that the transcriptomic features of the primary tumors yielded positive cancer grading and staging classification using DeepT2Vec and DeepC. The predictions for tumor grading and tumor staging are particularly interesting, which consists of the notion that tumor progression is largely dictated by gene activities of the primary tumors. The less accurate predictions could be due to the difficulties in distinguishing tumors with close grades and stages, the failure to identify metastatic events, and the potential of altered transcriptomic profiles between the primary tumors and the metastases, which would confound the training input. Given that the transcriptomic features are demonstrated informative for gene expression patterns, it is also possible that the discrete and subjective grading and staging system does not necessarily correlate with gene expression changes. For both scenarios, more training data are needed to improve performance.

### Fine-tune training the connected neural network of DeepT2Vec and classifier

As shown above, the transcriptomic TFVs generated from the DeepT2Vec feature-extracting network could be used in training classifiers for cancer diagnosis. It has been shown that one could potentially improve the performance by directly connecting the feature-extracting network to the classifier, and then training the connected network with forward and backward propagation between the original data input layer to the eventual output layer^1^. It is particularly useful if the unsupervised trained network, e.g., DeepT2Vec, could be used as pre-trained parameters for finetuning the connected network.

**Figure 6:**
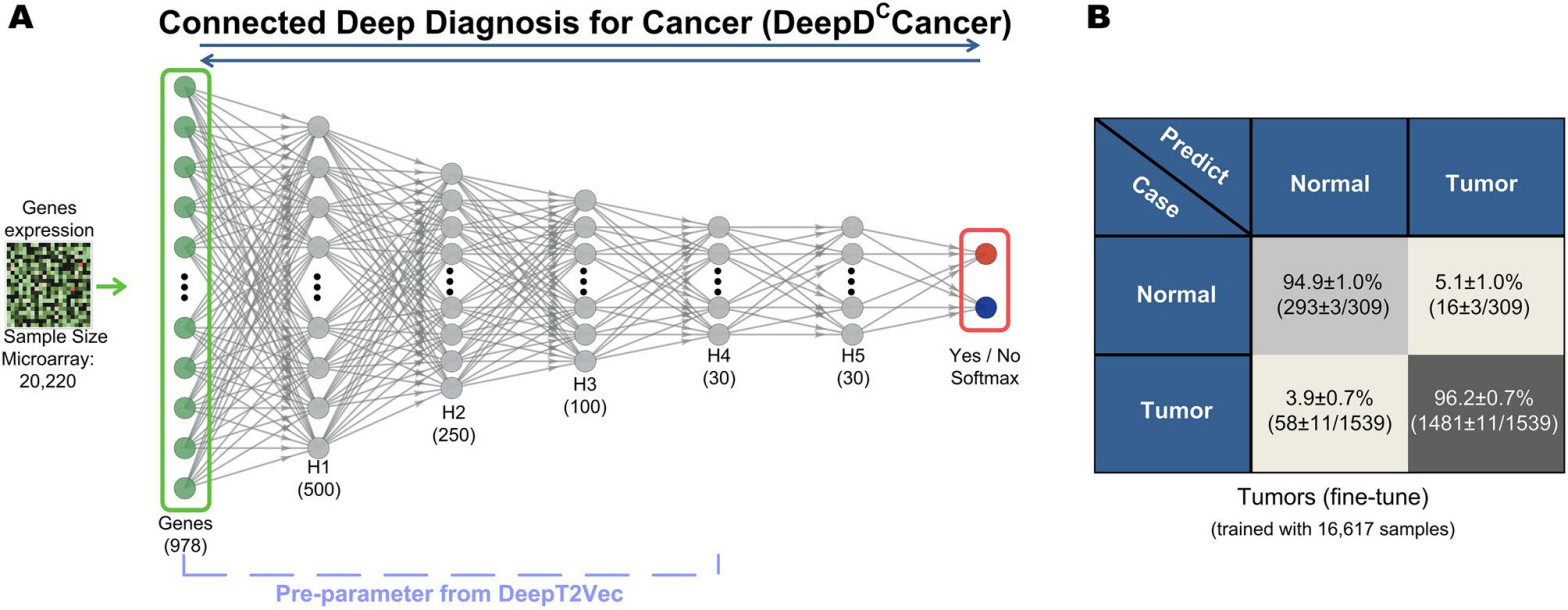
Semi-Supervised DeepD^C^Cancer network significantly improves diagnostic accuracy. (**A**) Schematic illustration of the connected DeepDCancer network, DeepD^C^Cancer, which couples the unsupervised DeepT2Vec with the supervised fully connected Softmax classifier. (**B**) Improved predictions by DeepD^C^Cancer after finetune training for Pan-Cancer and normal classification are shown.

We, therefore, generated a semi-supervised network for Pan-Cancer versus normal classification, called Connected Deep Diagnosis for Cancer (DeepD^C^Cancer; Fig. 6A), which consists of seven layers including the original input layer, five inter-layers, and the Softmax output layer. We used DeepT2Vec as the pre-trained parameters and conducted finetune training for this connected network using the same experimental dataset previously used. Similarly, 90% of the experimental data was used in training while 10% of the experimental data was used for testing, the SGD algorithm was used for optimization and the tenfold cross-validation was conducted. The parameters of all seven layers were modified through back-propagation during the finetune training. This resulted in a significant improvement for diagnosis: the classification accuracy was increased from 90 to 96% for Pan-Cancer and from 90 to 94% for normal tissues (Figs. 3A and 6B). These results further validate the applicability of deep autoencoder in feature extraction for analyzing complex transcriptomic data and its power for cancer diagnosis.

### Biological functional features identified by deep neural network learning

**Figure 7:**
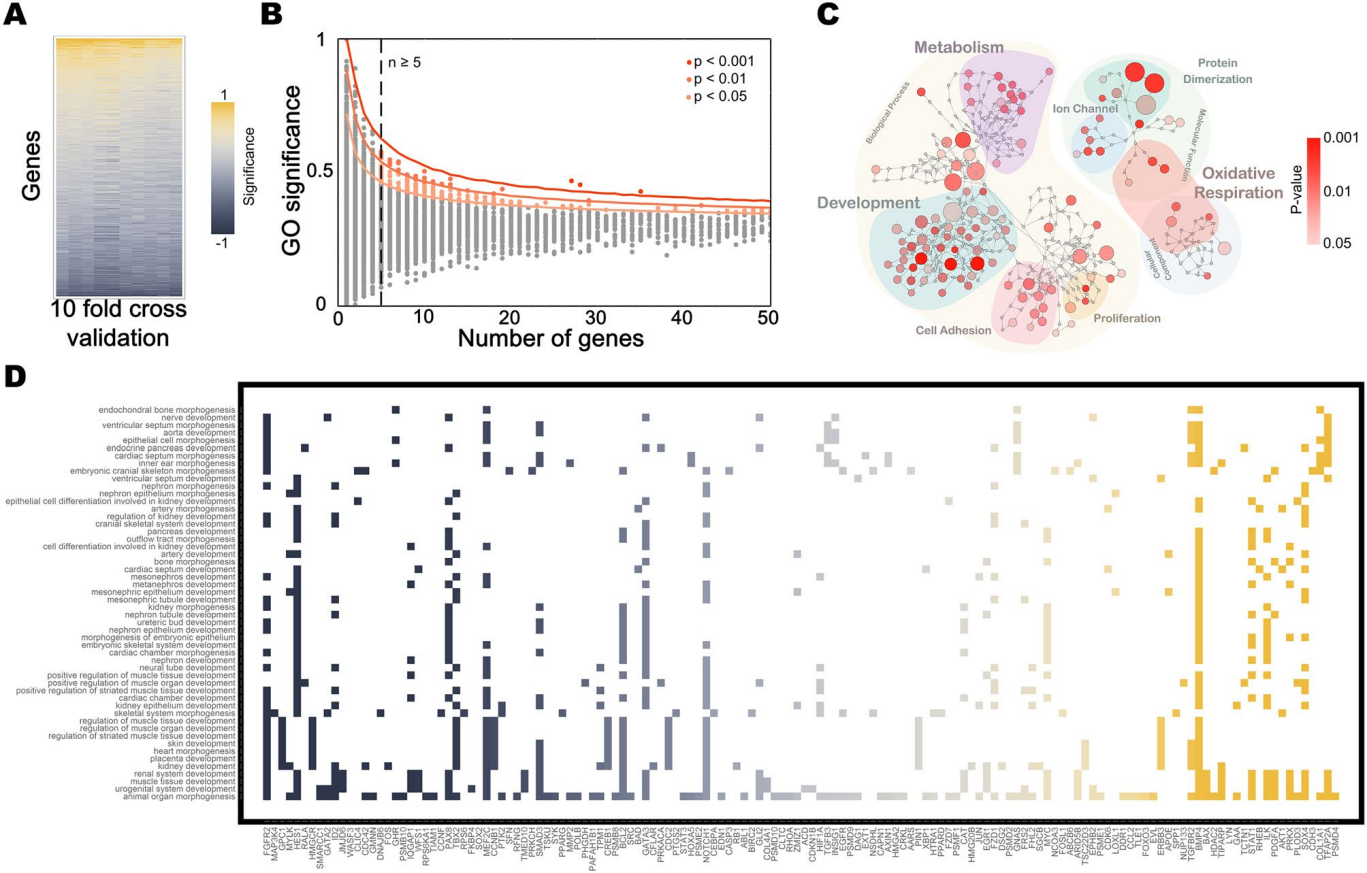
Identification of functional biological features from neural network. (**A**) The significance rank of the genes for predictability is illustrated in a heatmap, which is robust against tenfold cross validation. (**B**) The average significance of each GO function for connected DeepD^C^Cancer and the number of genes within them are plotted. Statistically significant GO functions were identified. (**C**) Graphic illustration of significant GO function groups and hierarchy relationship. Significant GO function groups are colored according to statistical significance and represented as circles of different sizes according to the number of genes. (**D**) Table illustration for genes identified in the GO function of *Development* in (**C**) and the specific developmental processes they participate in.

The analysis of transcriptomic data using a neural network for cancer prediction has been shown to be effective. Therefore, we were interested in learning what features were extracted and used for the classification. Given that the connected network achieved a better outcome, we further interrogated the weights of each connection in the connected network. To address this, we used the maximum activation algorithm, which interrogates the features extracted from the neural network for classification^34^. By using maximum positive or negative inputs randomly assigned to each neuron in the input layer to seek significant activation of each neuron in the next layer, we obtained maximal activation input patterns and generated an average value for those input patterns. The calculations were carried out for each layer, and the results from all the layers were then multiplied to calculate the contribution of individual input neuron to each output neuron. This analysis allowed us to calculate the significance of each gene on tumor or normal classification. One million randomly generated initial inputs were used to interrogate the connected neural network (“Materials and methods”). The genes of significant contribution identified by maximum activation analysis were robust against tenfold cross validation (Fig. 7A).

We did Gene Ontology (GO) enrichment analysis using DAVID (https://david.ncifcrf.gov) based on the contributions of the genes identified in the maximum activation analysis. 165/1217 GO annotations were identified as significant (p < 0.05) (Fig. 7B). Interestingly, about one-third of them (57/165) are associated with development or morphogenesis (Fig. 7C,D), consistent with previous findings suggesting the connection between cancer and development. Three smaller groups of identified genes are associated with metabolism, especially glucose metabolism and oxidative respiration (21/165), proliferation (7/165), and protein dimerization (6/165). Together, these results indicate that the deep neural network analysis of transcriptomic data achieved significant tumor classification prediction because of the recognition of patterns of tumor development.

## Discussion

Here we show the successful application of both unsupervised and supervised learning with a deep autoencoder and a classifier on a large collection of transcriptomic data from tumors and normal samples, which allows the extraction of transcriptomic features of the human genome and cancer diagnosis of high success rate. The successful application of deep learning to transcriptomic data other than imaging and text has multiple implications. Convolutional Neural Networks (CNNs), mimicking the mammalian visual system for local feature extraction and combinatorial information processing, have enabled the significant recent advances in image recognition. Similarly, Recurrent Neural Networks (RNNs) and its derivations are appropriately designed to extract features from sequential data and, therefore, have resulted in the successful development of natural language processing (NLP). However, much of the biomedical data do not share structural similarity with imaging or language. Gene expression data are high dimensional, sparse, nonlinear, and complex. Unlike images and language, transcriptomic data do not exhibit local or sequential correlation but are intrinsically organized and highly correlated. Here our results indicate that deep autoencoders, which are specialized to extract intrinsic features from complex data in a nonlinear way, can successfully extract important features from transcriptomic data. The deep autoencoder approach could be applicable to other molecular and physiological data in research, diagnosis, or therapeutics.

The approach to extract transcriptomic features by training a deep autoencoder in an unsupervised fashion on a large collection of transcriptomic data is highly successful. The resulting DeepT2Vec could convert complex transcriptomic data from each sample into a low dimensional vector, a 30-dimensional TFV. The success of the approach is evident by the finding that TFVs could be used to decode gene expression faithful to their original patterns. Furthermore, TFVs for samples from the same type of normal tissues are automatically clustered, while those from different tissue types are separated from each other. Interestingly, The TFVs for most of the tumor samples are gathered into a different space, away from the normal tissues, regardless of their tissue origins. The results indicate that tumors share cancer transcriptomic features, suggesting a common underlying molecular mechanism of carcinogenesis despite distinct tissue origins and oncogenic alterations. While it is a shared view that cancers exhibit common behaviors such as uncontrolled growth, this shared cancer transcriptomic profile, as defined by TFVs provides a molecular-based quantifiable measure that could be used in research and in the clinic.

Feature extraction without prior knowledge of the nature of genes provides the opportunity to a myriad of downstream analyses, including but not limited to clustering, data visualization, classification, and gene pathway analyses, especially across multiple datasets and systems. For cancer diagnosis, supervised training of a neural network classifier, DeepC, with TFVs from labeled samples yielded a high accuracy for Pan-Cancer (90%). Sensitivity analysis revealed that DeepT2Vec resulted from unsupervised training with a large dataset is powerful enough for analyzing data with relatively small sample size. This is further confirmed by analyses of tumors from the same tissue origin (brain, colon, breast, kidney, lung, skin, liver, prostate, ovary, or stomach), which have much fewer samples and yet are more biologically similar. Indeed, DeepDCancer has yielded even better predictions for specific types of tumors. Moreover, DeepD^C^Cancer further improved prediction accuracy for Pan-Cancer to 96%.

We envision the DeepD^C^Cancer framework as not only predictive but also comparable with human knowledge of cell biology (such as gene coexpression or signaling pathways). We examine this idea by conducting Gene Ontology analysis (Fig. 7) and compare the features learned from machine learning to the knowledge space of cell biology. The features “used” by the model to make accurate predictions highlight a collection of cellular modules. Those unmapped features learned from data can also be insightful as they might suggest previously undiscovered interactions or regulatory mechanisms. Secondly, while data-driven feature extraction is plausible, it is still optional and potentially beneficial to implement human prior knowledge of the system of interest. Prior knowledge can be added, for instance, as a regularization term in the overall loss function for neural network training so that the learning process can be steered to minimize both the reconstruction error and disagreement between prior information and information from extracted features. Furthermore, the recent development of transfer learning can also be applied to biological data, with the hypothesis that different cell systems (at least from the same organism) should share certain similarities. Such similarity could help, given a previously trained DeepD^C^Cancer model for one specific classification task, to train a second DeepD^C^Cancer model for another system of interest and to increase the accuracy meanwhile to decrease the size of samples required for such training.

We chose cancer diagnoses to be our proof-of-concept experiments without adding any other information into training. The success of such procedure suggest the potential usage of DeepD models in other disease types. Regarding cancer diagnosis specifically, many types of medical diagnoses are increasingly becoming machine-based and quantitative, and there have already been success, for example, using pathology images for diagnoses^4,5,8^. We also envision the merging of imaging-based and transcriptome-based diagnostic tools to raise the possibility for future AI-based assistance in cancer diagnosis, using big clinical data with multimodality. Transcriptome-based AI analysis also has additional advantages as it reveals molecular information that could have implications for signaling mechanisms, underlying mechanistic defects in diseases, and effects of therapeutic agents. Indeed, given that the transcriptome is the underlying driving force for biological and pathological changes, DeepT2Vec and TFVs could have broad applications. For example, identification of circulating tumor cells is critical for early detection and progression monitoring during therapy. Transcriptomic features are much easier to use than mutation- or morphology-based detection in naïve patients. Transcriptomic features could also serve as compound biomarkers for drug discovery and clinical trials, and provide quantitative indicators for future applications in personalized medicine.

In summary, the approach of unsupervised learning with a large transcriptome dataset, extracting inner relationships for gene expression regardless of specific biological process, has proven to be highly informative. Indeed, the resulting DeepT2Vec enables accurate predictions by classifiers trained by a small number of samples. This is evident in analyzing individual cancer types, which has yielded diagnostic accuracy above 95% using samples as few as in the hundreds. This is particularly important because many cases, such as rare diseases, require being able to work with a small sample set. Furthermore, as a powerful deep neural network with general transcriptomic features, DeepT2Vec, now that it has been produced, could be applied to many different settings with small sample sizes, reducing the cost for these analyses and easy to use for personal computers or mobile devices, which is particularly meaningful in clinical diagnosis.

## Materials and methods

### Data source and preprocessing

A set of 30,965 expression data of tumor and normal tissue samples were collected from Gene Expression across Normal and Tumor tissue (GENT) database [https://medical-genome.kribb.re.kr/GENT/] ^21^. Expression profiling of those samples was originally created with the Affymetrix® U133plus2 platform and was collected and uniformly normalized by GENT.

We filtered the dataset by genes using the LINCS L1000 list of m = 978 landmark genes. All the data (n = 30,965) were then preprocessed to remove outliers by the expression level of GAPDH, which was considered as a negative control and assumed to be uniform between samples (remove beyond 5% and 95% quantile of GAPDH expression, n = 27,930 after filtering). Samples with missing information about normal/cancer or tissue of origins and duplicate samples were also removed. After all the quality control filters, we ended up with a normalized dataset with in total 16,532 cancer samples and 3202 normal tissue samples.

We designed DeepD package in Python 3.7 using the Google Tensorflow framework (https://tensorflow.org) for all the computation procedures and the package is publicly available at https://github.com/DesmondYuan/deepD. The expression profiling was centralized to a distribution with mean = 0 and stand deviation = 1 on each gene and then on each cell. For stable training of neural networks, the entire data matrix was clipped by min = − 1 and max = 1 before being fed into autoencoders. 10% of the data were randomly selected as test dataset and not used for further training, and the remaining 90% was defined as experimental data. All the experimental data for training were subject to tenfold cross-validation whereas those data were split into 10 pieces and trained for 10 times where each piece served as validation dataset, respectivelyData preprocessing scripts were implemented using scikit-learn v0.23 and included in the DeepD.data package as a module.

### Network construction and model training

The neural networks described, including deep autoencoders and softmax classifiers, were established with DeepD.model module and trained with DeepD.train module. The deep autoencoder for transcriptomic feature extraction (DeepT2Vec) was trained with carefully finetuned hyperparameters, including activation function: tanh; iterations: 100,000; batch size: 1024; optimization algorithm: Adam optimizer; learning rate: 0.001; L1 regularization: 0.0001; L2 regularization: 0.01). Subject to tenfold cross-validation, 90% of experimental data were used for training, and the rest 10% experimental data were withheld as test set. Among the 90% experimental data, 70% (63% in total) were used for gradient descent and the other 30% (27% in total) used to monitor the training progress and early stopping when overfitting is detected the performance stops improving for a continuous period of time (patience: 1000 iterations). For Tumor Grading and Staging analyses, the same hyperparameters were applied. Model performance was evaluated by accuracies of each class (normal tissue/tumor); mean and standard deviation were calculated among all of the cross validation. For multiclass classification, all the cross validation models were combined in an unbiased fashion to make assembly predictions.

### Other state-of-the-art dimensionality reduction methods for comparison

The other dimensionality reduction methods are implemented using sklearn.decomposition package in Python (scikit-learn 0.23). Those methods are trained on the same 70/30% data partition and then connected to a Perceptron (shallow NN) classifier. For all of those methods, m = 30 components are calculated to match the vector width with Tp2vec.

### Accuracy and result visualization

Features identified through DeepT2Vec were visualized with unsupervised t-Distributed Stochastic Neighbor Embedding (t-SNE) using the Laurens van der Maaten’s t-SNE package^28^ and Matlab® R2013a. During training, real-time accuracy for supervised classification was calculated with the evaluation functions in DeepD.utils and was then used to monitor overfitting. The final accuracy was determined by selecting the point with the lowest MSE on the ROC curve. The average and standard deviation were calculated for tenfold cross validation. For multiclass classification (grades, T, N, M, stages), separate one-versus-rest classifiers for each class were trained independently.

## Supplementary information

Supplementary Information
