# Supplementary material for: Unsupervised and supervised learning with neural network for human transcriptome analysis and cancer diagnosis
Source: Sci Rep. 2020 Nov 5;10:19106. doi: 10.1038/s41598-020-75715-0 (PMC7644700; doi:10.1038/s41598-020-75715-0)
Supplement: Supplementary file 1 — Supplementary Information [file 41598_2020_75715_MOESM1_ESM.pdf]

Unsupervised and Supervised Learning with Neural Network for Human Transcriptome Analysis and Cancer Diagnosis

Bo Yuan2,3†, Dong Yang1,2†, Bonnie EG Rothberg4, Hao Chang2, Tian Xu1,2*

[1]Westlake Institute for Advanced Study, Westlake University, Hangzhou, China

[2] Howard Hughes Medical Institute, Department of Genetics, Yale Cancer Center, Yale University School of Medicine, 295 Congress Avenue, New Haven CT 06510, USA.

[3] Zhiyuan College, Shanghai Jiao Tong University, Shanghai, China

[4] Medical Oncology, Department of Internal Medicine, Yale Cancer Center, Yale University School of Medicine

*Corresponding author: Email: tian.xu@yale.edu.

†Equal contribution.

The authors declare no potential conflicts of interest.

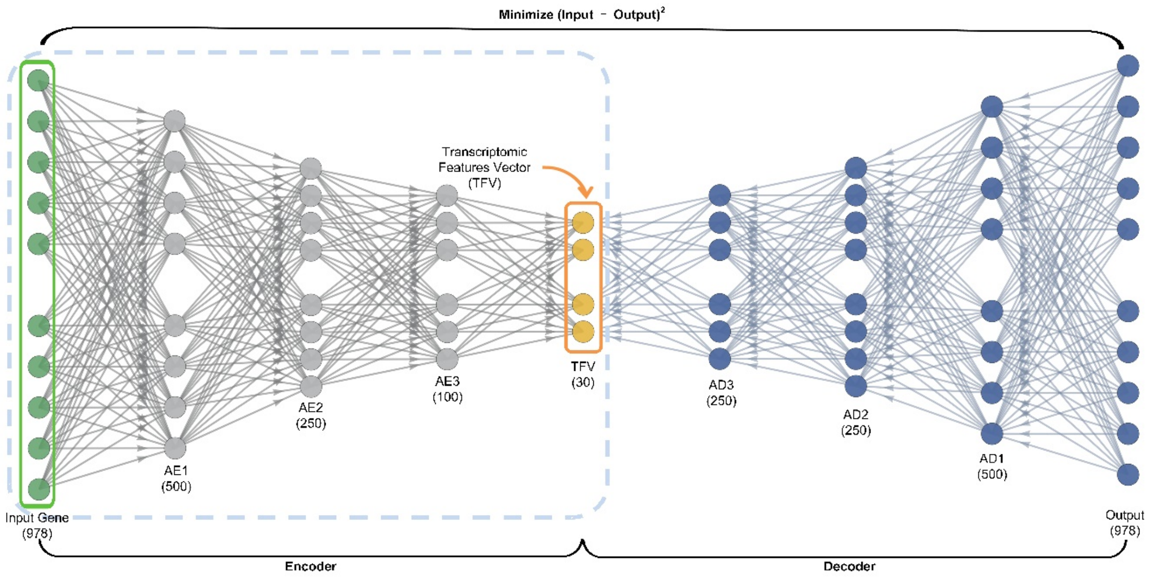

Fig S1. The architecture of deep autoencoder.

The autoencoder is an unsupervised neural network for the task of representation learning, which consists of encoder and decoder. The training process is to minimize the loss between the input and output. After training the encoder, called DeepT2Vec, is used to compress genes expression pattern into 30-dimension TFV, and the decoder is used to evaluate reconstruction performance.

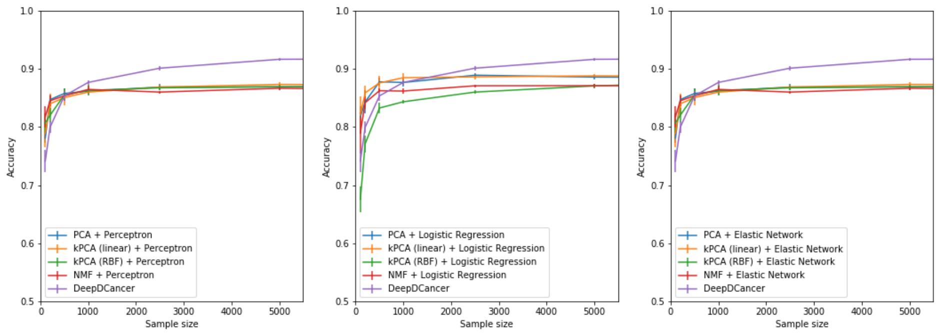

Fig S2. Accuracy comparison between DeepDCancer and the other four state-of-the-art methods.

PCA: principal components analysis, kPCA (linear): linear kernel PCA, kPCA(RBF): RBF kernel PCA, and NMF: Non-negative matrix factorization. Elastic Network, Logistic Regression, and Perceptron were trained respectively using different features extracted by other dimensionality reduction methods to compare the predictive power with DeepDCancer.
